# Serum Antibodies Against Simian Virus 40 Large T Antigen, the Viral Oncoprotein, in Osteosarcoma Patients

**DOI:** 10.3389/fcell.2018.00064

**Published:** 2018-06-22

**Authors:** Elisa Mazzoni, Ilaria Bononi, Maria S. Benassi, Piero Picci, Elena Torreggiani, Marika Rossini, Andrea Simioli, Maria V. Casali, Paola Rizzo, Mauro Tognon, Fernanda Martini

**Affiliations:** ^1^Laboratories of Cell Biology and Molecular Genetics, Section of Pathology, Oncology and Experimental Biology, Department of Morphology, Surgery and Experimental Medicine, University of Ferrara, Ferrara, Italy; ^2^Laboratory of Experimental Oncology, Rizzoli Orthopedic Institute, Bologna, Italy; ^3^Headquarter Department, State Hospital, Republic of San Marino, San Marino, San Marino; ^4^Maria Cecilia Hospital, GVM Care & Research, E.S. Health Science Foundation, Cotignola, Italy; ^5^Laboratory for Technologies of Advanced Therapies (LTTA), University of Ferrara, Ferrara, Italy

**Keywords:** osteosarcoma, SV40, Tag, antibody, prevalence

## Abstract

Human osteosarcoma (OS) is a rare human cancer, mostly occurring in children and adolescents. Simian virus 40 (SV40 = Macaca mulatta polyomavirus 1) sequences have been detected in different human cancers, including osteosarcoma. SV40 is an oncogenic virus *in vivo*, whereas it transforms different kinds of mammalian cells, as well as distinct human cell types. SV40 injected in rodents induces tumors of different histotypes, such as bone and brain tumors. Herein, the association between OS and SV40 large T antigen (Tag) was studied by employing indirect ELISAs using synthetic peptides that mimic different epitopes of the SV40 Tag, the viral oncoprotein. Indirect ELISAs were used to detect serum IgG antibodies against this oncogenic virus in samples from OS patients. Controls were sera from healthy subjects (HS) and oncological patients affect by breast cancer (BC), which is not associated with SV40. It turned out that sera of OS patients had a higher prevalence of SV40 Tag antibodies, 35%, compared to HS, 20% and BC, 19%, respectively. The different prevalence of SV40 Tag antibodies revealed in OS vs. HS and vs. BC is statistically significant with *P* < 0.05 and *P* < 0.01, respectively. Our immunological data indicate a significantly higher prevalence of antibodies against SV40 Tag epitopes in serum samples from OS patients compared to HS and BC, the controls. These results suggest an association between OS and SV40 Tag, indicating that this oncogenic virus may be a cofactor in OS development.

## Introduction

Human osteosarcoma (OS), which derives from mesenchymal cells, is a common malignancy of the bone (Li et al., 2009). Its incidence rates and 95% confidence intervals in different populations are 4.0 (3.5–4.6) and 5.0 (4.6–5.6) per year/million, in the range 0.1–14 years old, and 0.1–19 years old, respectively (Ottaviani and Jaffe, 2009; Duchman et al., 2015). In recent years, the introduction of new compounds, including biological drugs, to OS therapy has improved the clinical outcome of this bone cancer, while reducing patient death rate (Savage and Mirabello, 2017). However, metastasis, recurrences and multiple drugs resistance may occur in OS affected patients (Omer et al., 2017; Fidler et al., 2018). While factors responsible for OS onset/progression are not completely known, chemical, physical and biological carcinogenic agents are believed to play a key role in targeting cellular genome/genes, as with other human tumors (Gianferante et al., 2017; Savage and Mirabello, 2017). Clastogenic, mutagenic and carcinogenic agents cause chromosome aberrations and gene mutations (Gianferante et al., 2017; Savage and Mirabello, 2017). Indeed, many different genome alterations have been detected in OS, such as chromosome deletions, translocation, duplications, alongside other molecular and point mutations (Yang et al., 2017).

Among environmental infectious agents, viruses with oncogenic potential, such as polyomaviruses, have frequently been advocated as causal agents in OS onset (Nelson, 2001; Klein et al., 2002; Barbanti-Brodano et al., 2004; WHO, 2013). Tumorigenic polyomaviruses have found to be associated with OS, as well as other human cancers, despite being highly spread in the general population (Jasani et al., 2001; Rinaldo et al., 2003; Mazzoni et al., 2015). Their oncogenic potential is linked to the expression of two main viral oncogenes, named large T antigen (Tag) and small t antigen (tag). These two encoded viral oncoproteins of the early transcription unit, act like activated oncogenes. Their transforming abilities can be expressed mainly in non-permissive or semi-permissive cells where the polyomavirus cannot fully multiply (Martini et al., 2007). Specific human cells resistant to the virus multiplication activity are prone to transformation since Tag/tag viral oncoproteins, expressed into the cell nucleus, bind and abolish the cellular gene products p53 and pRB tumor suppressor families (Comerford et al., 2012; Batisse-Lignier et al., 2017).

In the absence of these tumor suppressor activities, cells multiply accumulating an impressive amount of mutations in their DNA, thus facilitating the multistep process of cell transformation (Comerford et al., 2012; Batisse-Lignier et al., 2017). *In vivo*, when the genome acquires too many mutations, the risk of tumor onset/progression increases dramatically (Batisse-Lignier et al., 2017). Simian virus 40 (SV40 = Macaca mulatta polyomavirus 1) (Polyomaviridae Study Group of the International Committee on Taxonomy of Viruses et al., 2016; Moens et al., 2017) a polyomavirus with oncogenic potential, is able to induce tumors of different histotypes, including osteosarcomas and sarcomas in inoculated rodents in experimental conditions (Marton et al., 2000; Schell et al., 2000; Vilchez et al., 2004). Moreover, rodents injected with recombinant plasmids expressing SV40 Tag under specific tissue promoters also develop osteosarcoma and sarcoma (Diamandopoulos, 1972; Knowles et al., 1990; Wilkie et al., 1994). SV40 DNA sequences and Tag expression, both at mRNA and protein levels have been reported in human osteosarcoma by many investigators (Heinsohn et al., 2000, 2009; Yamamoto et al., 2000; Malkin et al., 2001; Ziadi et al., 2012). Other studies reported negative data on SV40 footprints in human tumors (Carter et al., 2003). More recently with the development of a specific immunologic assay with synthetic peptides as mimotopes, which mimic SV40 viral capsid protein (VP) epitopes, IgG antibodies reacting to these viral antigens were detected at higher prevalence in OS patient sera compared to controls (Mazzoni et al., 2015). Negative serological data have also been reported in researches into an association between human tumors and SV40 (Martini et al., 2013; WHO, 2013).

In this study, a new serum collection from OS patients was investigated. Serum samples were analyzed with indirect ELISAs, which employ mimotopes of SV40 Tag, the viral oncoprotein. Sera from OS patients and healthy subjects (HS), as well as serum samples of patients with breast carcinoma (BC), were tested for their reactivity to SV40 Tag epitopes, represented by two synthetic peptides, mimicking Tag antigens.

## Materials and methods

### Serum samples

Sera from OS affeçcted patients were harvested between 2002 and 2012 at the Rizzoli Orthopedic Institute, Bologna, Italy. These samples were collected from OS patients (*n* = 249) with a histologically proven diagnosis of different types of OS localized in distinct anatomical sites (Table 1). Sera from HS (*n* = 247) were obtained from the Clinical Laboratory Analysis, the Delta Hospital of Ferrara, Italy and the State Hospital, Republic of San Marino. Healthy subjects were of similar mean age of 21 years old (4–76 years) and gender (47% male) of the oncologic patients with a mean age of 22 years old (6–76 years) and 56% male (Table 1). Additional serum samples were from patients affected by breast cancer (BC) (Martini et al., 2013) (**Table 4**). Written informed consent was obtained from adult patients/individuals, whereas for children and young adolescents < 18 years old, the written informed consent was provided by parents. Anonymously collected sera were coded with indications of age, gender, and pathology. The County Ethical Committee of Ferrara, Italy, approved the project, study number 151078.

**Table 1:** Osteosarcoma subtypes and SV40 Tag–positive samples.

**Patient/Subject**	**Number of serum samples**	**Number of Tag A+D positive samples (%)**
**OS TYPES AND SUBTYPES**		
Conventional Osteosarcoma	243	86 (36)
Osteoblastic	193	71 (37)
Chondroblastic	20	7 (35)
Fibroblastic	25	8 (32)
Telangiectatic	5	0
Multicentric	1	1 (100)
Parosteal	3	0
Osteosarcoma in Paget's disease	1	0
OS in other benign damage	1	0
OS Total sera	249	87 (35)^*^
HS Total sera	247	56 (23)

*OS, osteosarcoma; HS, healthy subject; SV40, simian virus 40. Human sera were from OS patients and HS*.

The prevalence of SV40 Tag antibodies in all OS patients is statistically significant, higher than in HS (^*^P < 0.01).

*The prevalence of SV40 Tag antibodies in sera from patients affected by distinct OS subtypes does not differ statistically among them (P > 0.05)*.

*P-values were determined using the Chi-square*.

### Indirect elisa

The indirect ELISA employed herein has been recently reported (Tognon et al., 2016). Briefly, mimotopes known as Tag A and D were employed in indirect ELISAs to detect SV40 Tag serum IgG antibodies.

#### Coating phase

Immunological plates (Nunc-immuno plate PolySorp, Thermo Fisher Scientific, Milan, Italy) were coated with 5 μg of the Tag A or Tag D peptide (the a.a. sequences are reported below) in each well, diluted in 100 μl of Coating Buffer 1X, pH 9.6 (Candor Bioscience, Wangen, Germany). Coated peptides were left at 4°C for 16 h. Amino acid sequences of the two Tag A and Tag D synthetic peptides from a.a. residues 669–689 (21 a.a.) and from 659–682 (24 a.a.), respectively, are as follows:

Tag A: NH2-G S F Q A P Q S S Q S V H D H N Q P Y H I-COOH (Tag a.a. 669–689);
Tag D: NH2-H E T G I D S Q S Q G S F Q A P Q S S Q S V H D-COOH (Tag a.a. 659–682).

These two mimotopes were purchased from UFPeptides s.r.l., Ferrara, Italy.

#### Blocking phase

Coated peptides were rinsed twice with Tris-based Washing Buffer (Candor Bioscience, Wangen Germany). The blocking solution (200 μl), containing the casein and Tween detergent (Candor Bioscience, Wangen, Germany), pH 7.2, was added to each well.

#### Sera addition

Wells were rinsed three times with Tris-based Washing Buffer (Candor Bioscience, Wangen, Germany). Then, 1/20 diluted sera were added to wells.

#### Control samples

Positive controls were represented by hyperimmune rabbit serum containing anti-SV40 Tag antibodies (diluted 1:100) (Tognon et al., 2016); (i) negative controls were hyperimmune sera with anti-BKPyV and anti-JCPyV antibodies (diluted 1: 100); (ii) three human sera found to be SV40-negative in a previous report (Tognon et al., 2016) (iii) SV40 unrelated human neuropeptide S (hNPS), a.a sequence SFRNGVGTGMKKTSFQRAKS (Guerrini et al., 2010; Tognon et al., 2016).

#### Sera under analysis

Sera from OS, BC patients and HS were diluted in Low Cross-Buffer pH 7.2 (Candor Bioscience, Wangen, Germany) initially at 1:20 and then for the IgG titer evaluation at dilutions up to 1:320. Additional controls contained secondary antibody only or were wells void of both primary and secondary antibodies. Immuno-complex reactions were carried out at 37°C for 90 min. Each sample was analyzed three times in duplicate wells.

#### Secondary antibody addition

Wells were rinsed twice with the Washing Buffer (Candor Bioscience, Wangen, Germany). Then, the secondary antibody was added to each sample. This solution contained goat anti-human or goat anti-rabbit IgG heavy (H) and light (L) chain-specific peroxidaseconjugate (Calbiochem-Merck, Darmstadt, Germany).

#### Optical density reading and cutoff

At the end of the incubation period, the plates were rinsed three times with Washing Buffer and then treated with 100 μl of 2,2′-azino-bis 3-ethylbenzthiazoline- 6-sulfonic acid (ABTS) (Sigma-Aldrich, Milan) in buffer solution (9.1 mM ABTS; pH 5.0), which reacted with the peroxidase enzyme to yield the color reaction. The plate was then read spectrophotometrically (Thermo Electron Corp., model Multiskan EX, Finland) at a wavelength (λ) of 405 nm. This optical density (OD) reading reflected the extent of immunocomplexes formed by the presence of specific antibodies, which bound to the SV40 synthetic peptide/epitopes/mimotopes. The three SV40 negative control sera were selected from samples below the cut-off value determined with second–degree polynomial regression by plotting the ranked net OD individual values for each peptide. A tendency curve was drawn from a second–degree polynomial regression for Tag A peptide and Tag D peptide, as published for MCPyV and BKPyV virus-like particles (VLPs) (Touzé et al., 2010). Our OD data/analysis revealed an inflection point corresponding to 0.18 for each peptide (Tognon et al., 2016).

### Total IgG

Total IgG concentrations in serum samples of OS patients (*n* = 50) were assessed using the commercial kit “Human total IgG Platinum Enzyme-Linked Immunosorbent Assay (ELISA)” according to the manufacturer's instructions (eBioscience) The ELISA plate was read spectrophotometrically (Thermo Electron Corp., model Multiskan EX, Finland) at a wavelength (λ) of 450 nm. The reference intervals for healthy adults was IgG 700–1,600 mg/dL (Dati et al., 1996; Gonzalez-Quintela et al., 2008). The lower threshold for detection of IgG with this method is 2 pg/ml.

### Statistical analysis

The prevalence of SV40 Tag-positive samples from OS patients and controls was determined using Chi-square with Yates' correction. Intra-run and inter-run variability of OD values and IgG values were analyzed using the Unpaired *t*-test. The serologic profile of the reactivity to SV40 Tag mimotopes was statistically analyzed using the Anova and Tukey's multiple comparisons test. All computational analyses were performed with Prism 6.0 (Graph- Pad software, San Diego, CA). For all tests, *P* was considered to be statistically significant when *P* < 0.05. The total IgG values were statistically analyzed using Anova and Newman-Keuls multiple comparisons test.

## Results

### IgG antibodies reacting to SV40 tag mimotopes in sera from osteosarcoma affected patients

Serum samples from OS patients (Table 1), diluted 1:20, were assayed by indirect ELISAs with SV40 Tag mimotopes A and D. These sera reacted to mimotope A with a prevalence of 38%, whereas samples from heathy subjects (HS) reached a prevalence of 26%. The different prevalence between the two cohorts is statistically significant (*P* < 0.01) (Tables 1, 2).

**Table 2:** Prevalence of IgG antibodies reacting to SV40 Tag mimotopes in serum samples from OS patients, treated and untreated, compared to HS.

**Serum**		**Number of patients/individuals**	**Mean age (Range) years**	**Male, %**	**Number of SV40 Tag- positive samples (%)**		
					**Tag A**	**Tag D**	**Tag (A+D)**
OS		249	22 (6–76)	56	94 (38)	99 (40)	87 (35)^*^
	Treated	166	21 (6–62)	55	61 (37)	67 (41)	56 (34)°
	Untreated	83	25 (8–76)	58	33 (40)	32 (39)	31 (37)^§^
Outcome							
	No metastasis	83	25 (8–76)	58	54 (36)	56 (38)	49 (33)
	Metastasis	100	21 (6–72)	53	40 (40)	43 (43)	38 (38)
	Dead	105	24 (7–76)	66	42 (40)	45 (43)	37 (35)
HS		247	21 (4–76)	47	63 (26)	63 (26)	56 (23)

OS, osteosarcoma; HS, healthy subjects; SV40, simian virus 40. The different prevalence of SV40 Tag antibodies in osteosarcoma OS patients was statistically significant compared to HS (^*^P < 0.01).

*The different prevalence of SV40 Tag antibodies in OS patients, either treated with chemotherapy or without treatment, were statistically significant compared to HS (^§°^P < 0.05)*.

*P values were determined using the Chi-square with Yates' correction*.

Then, the same sera were assayed with the other SV40 Tag mimotope, represented by the peptide D. In this instance, the prevalence of IgG antibodies from OS patients reacting to this antigen was 40%. Sera from HS tested SV40 Tag peptide D positive with a prevalence of 26%. The difference between the two cohorts, OS vs. HS, is statistically significant with a *P-*value < 0.01 (Table 2).

In our indirect ELISA only serum samples reacting with both synthetic peptides A and D were accepted as SV40 Tag-positive.

The prevalence of all OS sera tested positive for peptides A and D reached 35%, while the prevalence detected in sera from HS was 23% (Table 2). These data indicate that the higher prevalence of SV40 Tag-positive detected in samples from OS patients, using the *X*^2^ test with Yates's correction, is statistically significant compared to HS (^*^P < 0.01) (Table 2).

It is worth noting that in previous immunological studies with HS sera, in which the same indirect ELISA with SV40 Tag mimotopes A and D was employed, the prevalence of SV40 Tag antibodies did not differ substantially in the different cohorts of HS (HS 1–4) analyzed, being in the range of 19–22% (Tognon et al., 2016; Mazzoni et al., 2017a,b,c). In this study, the prevalence in HS was 23%. The little difference in prevalence is probably due to the distinct age of HS (Table 3).

**Table 3:** Prevalence of IgG antibodies in sera from HS, reacting to SV40 Tag mimotopes A and D reported in different studies, and OS patients analyzed in this investigation.

**Human serum**	**Articles**	**Age years; Mean age range**	**Number of SV40 Tag A+D positive samples/samples analyzed (%)**	***P*-Value**
OS	This study	22; 6–76	87/249 (35)	
HS	This study	21; 4–76	56/247 (23)	<0.01
HS1	Tognon et al., 2016	44;18–65	138/704 (20)	<0.0001
HS2	Mazzoni et al., 2017b	78; 66–100	60/273 (22)	<0.01
HS3	Mazzoni et al., 2017b	57; 22–91	49/254 (19)	<0.01
HS4	Mazzoni et al., 2017b	30; 18–40	36/180 (20)	<0.01

*Human sera were from OS patients and HS. The prevalence of SV40 Tag antibodies among the cohorts of HS 1–4 were reported in previous articles, whereas the prevalence of SV40 Tag antibodies in OS sera were analyzed in this study, together with the new cohort of HS*.

*The prevalence of SV40 Tag antibodies in OS sera is statistically significant compared to HS and HS 1-4 (P < 0.01 and P < 0.0001)*.

*Statistical analysis was performed using the Chi Square with Yates'correction*.

**Figure 1:**
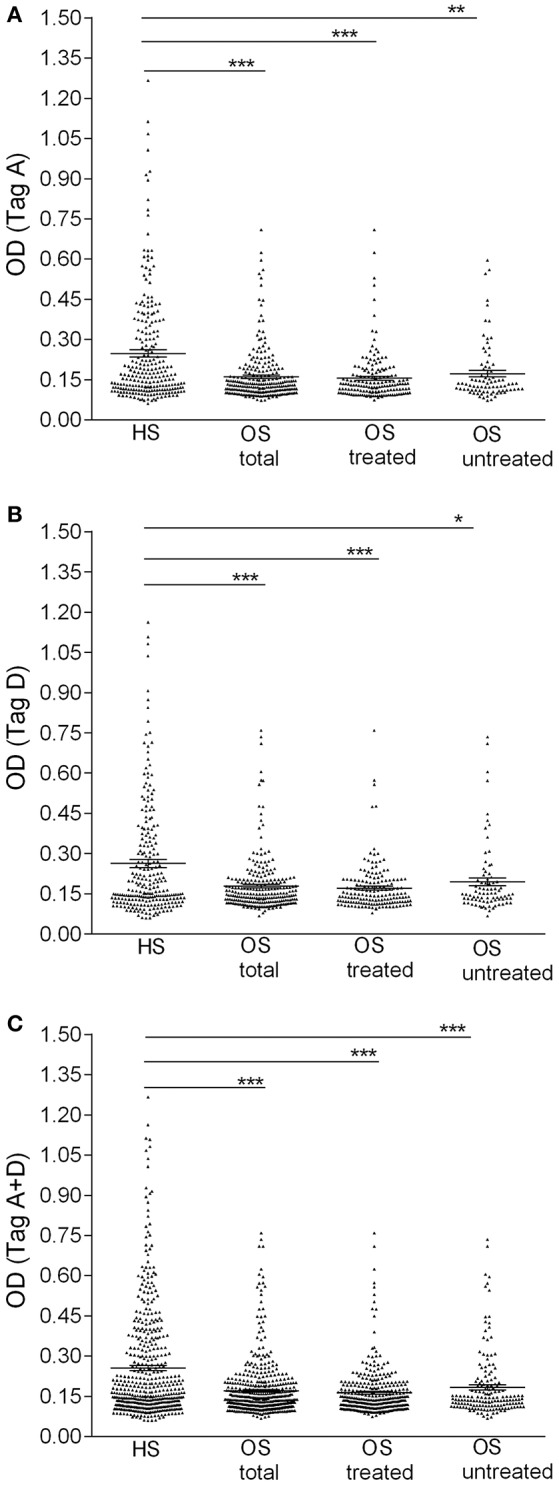
Serologic profile of serum antibody reactivity to SV40 Tag. Mimotopes A **(A)**, mimotope D **(B)**, and Tag mimotopes A + D **(C)**. Immunologic data are from OS patients (OS total), OS patients treated with chemotherapeutic drugs (OS treated), OS without treatment (OS untreated) and healthy subjects (HS). Results are presented as OD values. In the scatter dot plotting, each plot represents the dispersion of individual sample OD values to a mean level, indicated by the long horizontal line inside the scatter with the standard error of the mean (SEM) marked by a short horizontal line for each age group. Data were analyzed with one-way Anova analysis and Tukey's multiple comparisons test (OD mean, 95% CI). **(A)** The mean OD of sera against SV40 mimotope Tag A (TagA ± SEM) in all OS patients (0.16 ± 0.006), in OS treated (0.15 ± 0.007) and in OS untreated (0.17 ± 0.012) was significantly lower than that of HS sera (0.25 ± 0.013). **(B)** The mean OD of sera against SV40 mimotope Tag D (TagD ± SEM) in all OS patients (0.18 ± 0.007), in OS treated (0.17 ± 0.006), and in OS without treatment (0.19 ± 0.014) was significantly lower than HS subjects (0.26 ± 0.015). **(C)** OD value for antibodies against SV40 mimotopes both peptides (Tag A and Tag D) in all OS patients (TagA + TagD ± SEM) (0.17 ± 0.005), in OS treated (0.16 ± 0.005) and in OS untreated (0.18 ± 0.01) was lower than HS sera (0.26 ± 0.01). The average OD values between total OS, OS treated and OS without treatment are not statistically different (*P* > 0.05). ^***^P < 0.0001; ^**^P < 0.001; ^*^P < 0.01.

Serologic profiles are shown in Figure 1. The prevalence of SV40 Tag-positive antibodies in serum samples from OS patients with or without metastasis did not differ (*P* > 0.05). In addition, in OS patients, found to be SV40 Tag-positive, the prevalence of metastasis (38/100 = 38%) was not statistically different compared to SV40 Tag-positive OS patients, without metastasis (49/149 = 33%, *P* > 0.05). Moreover, the prevalence of SV40 Tag-positive (37/105 = 35%) among OS patients, who died of this tumor, is similar to that revealed in survived patients, who became OS negative (ned) (50/144 = 35%, *P* > 0.05) (Table 2). These results indicate that SV40 does not increase the prevalence of death/metastasis in OS patients with serum antibodies against SV40 Tag.

As an additional control, serum samples (*n* = 78) from breast cancer patients were tested by indirect ELISA (Martini et al., 2013). It is worth recalling that BC is a human carcinoma, which has not been found linked to the SV40 infection (Martini et al., 2013). BC serum IgG antibodies against SV40 Tag reached a prevalence of 19%. SV40 Tag antibodies from OS patients had a statistically significant prevalence compared to BC patients (35 vs. 19%; *P* < 0.01), while the prevalence detected in HS and BC was not statistically different (*P* > 0.05) (Table 4).

**Table 4:** Prevalence of IgG antibodies reacting to SV40 Tag mimotopes in serum samples from OS patients, compared to HS and BC.

**Serum**	**Number of Patients/Individuals**	**Mean Age (Range) Years**	**Male %**	**Number of SV40 Tag-positive samples (%)**		
				**Tag A**	**Tag D**	**Tag A+D**
OS	249	22 (6–76)	56	94 (38)	99 (40)	87 (35)
HS	247	21 (4–76)	47	63 (26)	63 (26)	56 (23)^*^
BC	78	42 (25–69)	0	15 (19)	17 (22)	15 (19)°

*OS, osteosarcoma affected patients; HS, healthy subjects; BC, breast cancer affected patients; IgG, immunoglobulin G; SV40, Simian virus 40; Tag, large T antigen; Tag A and Tag D, synthetic peptides/mimotopes employed in indirect ELISA to detect SV40 Tag antibodies*.

The different prevalence of SV40 antibodies in OS sera compared to HS was statistically significant (^*^P < 0.01).

*The prevalence of SV40 antibodies in OS sera differs statistically from BC (°P < 0.01)*.

*P values were determined using the Chi-square with Yates' correction*.

**Figure 2:**
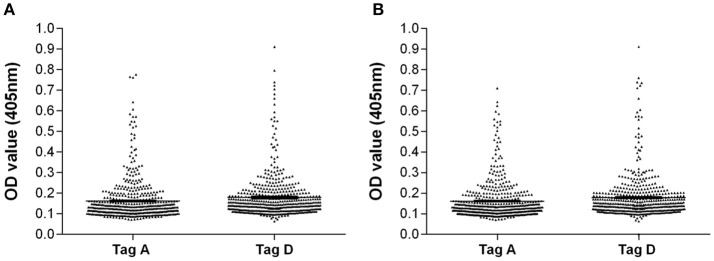
Intra-run and inter-run variability of OD values of serum antibody reactivity to Tag A and D peptides in OS patients. Data are presented as scatter dot plot of OD readings at λ 405 nm, mean and standard error of the mean (SEM) marked by short horizontal lines for each peptide. **(A)** OD value variability, intra-run. Tag A: mean = 0.16, SEM = 0.005; Tag D: mean = 0.18; SEM = 0.005. **(B)** OD value variability, inter-run. Tag A: mean = 0.16, SEM = 0.004; Tag D: mean = 0.18, SEM = 0.005.

In inter-run and intra-run tests, the variability of OD values of antibody reactivity to Tag A and D peptides in sera from OS patients were not statistically significant. Serologic profiles are shown in Figure 2.

### Titer of IgG antibodies against SV40 tag in positive OS sera

**Figure 3:**
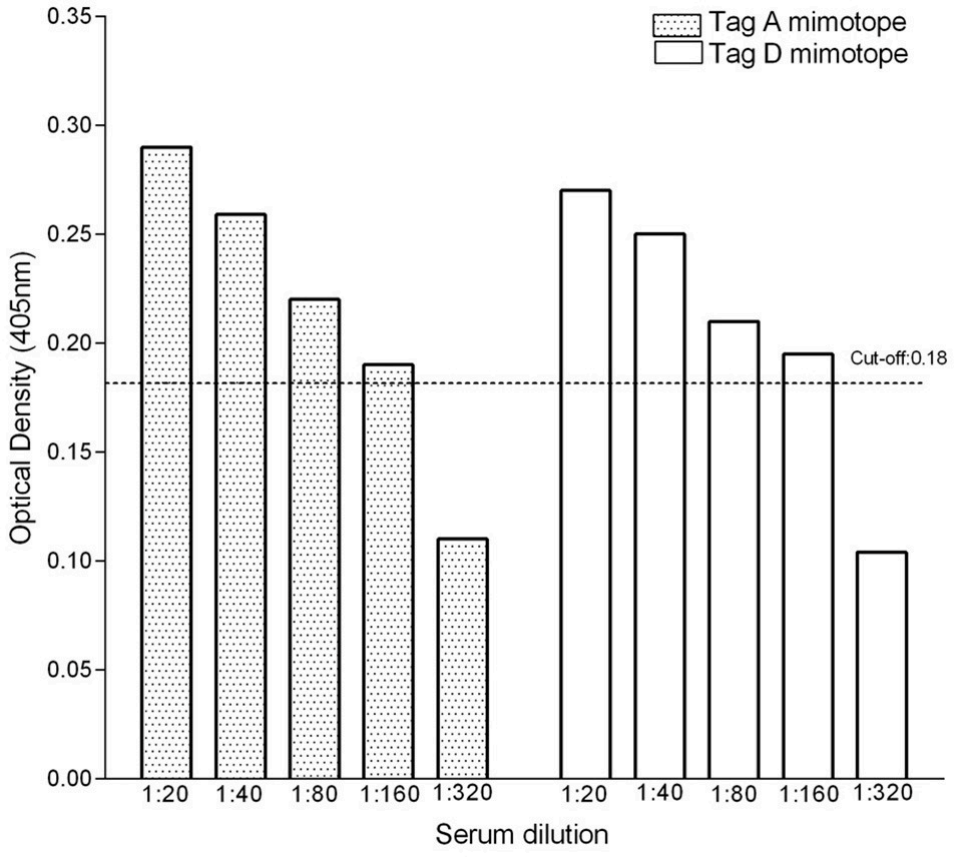
Titer of IgG antibodies against SV40 Tag in OS-positive sera. To verify the antibody titer, sera (*n* = 4) from OS patients found to be SV40 Tag-positive for both mimotopes A and D, were serially diluted from 1/20 to 1/320 and further investigated by indirect ELISAs. These assays indicated that sera carrying IgG antibodies against SV40 Tag remain positive at 1/160 dilution.

OS sera (*n* = 4), which were found to be SV40-positive for both Tag A and Tag D peptides, with an OD in the 0.29–0.27 range, were serially diluted from 1/20 to 1/320 to determine their Tag antibody titer. These sera contained antibodies against SV40 Tag, which remained detectable at 1/160 dilution (Figure 3). This result indicates that the titer of SV40 Tag antibodies, in positive sera from OS patients, does not greatly differ for the two A and D mimotopes. The reproducibility of the results was assessed with three replica experiments carried out by independent operators with no data variability.

### Serum antibodies against SV40 tag mimotopes in patients with different types of osteosarcoma, treated with chemotherapeutic drugs

OS are heterogeneous cancers, which are classified into many subtypes. In our study, OS were represented by conventional grade 4 OS including osteoblastic (*n* = 193), chondroblastic (*n* = 20) and fibroblastic (*n* = 25) subtypes. In addition, we analyzed telangiectatic (*n* = 5), multicentric (*n* = 1), parosteal (*n* = 3), osteosarcoma in Paget's disease (*n* = 1), and other benign OS damage (*n* = 1), (Table 1). Then, statistical analyses were carried out on the total number of OS, divided into two different cohorts, OS treated with chemotherapeutic drugs (OS treated) and OS without treatment (OS untreated). Treated OS patients had a mean age of 21 years old, with a range of 6–62 years old, whereas untreated osteosarcoma patients had a mean age of 25 years old, with a range of 8–76 years old. The presence of SV40 Tag antibodies in these two different cohorts of OS patients was observed with a prevalence of 34% (56/166) and 37% (31/83), in OS treated and OS untreated patients, respectively. The prevalence between OS treated (34%) and OS untreated (37%) was not statistically different (*P* > 0.05) (Table 3), while the different prevalence of SV40 Tag antibodies in OS patients subjected to chemotherapy and OS patients without chemotherapy were statistically significant compared to the cohort of HS (^◦§^*P* < 0.05) *X*^2^ with Yates' correction (Table 2).

### Profiles of sera from patients with OS treated with chemotherapeutic drugs and healthy subjects

Immunological OD values obtained with OS serum samples, whether treated with chemotherapy or not, tested positive for SV40 Tag antigens, were lower than in HS. Indeed, OD values for the single mimotope A or mimotope D, and both peptides A + D in OS patients, untreated and treated with chemotherapy, were lower than that of HS. Serologic profiles are shown in Figure 1. Interestingly, OD values detected in treated OS and untreated OS, were not statistically different (*P* > 0.05).

### Distribution of serum immunoglobulin levels in osteosarcoma affected patients

**Figure 4:**
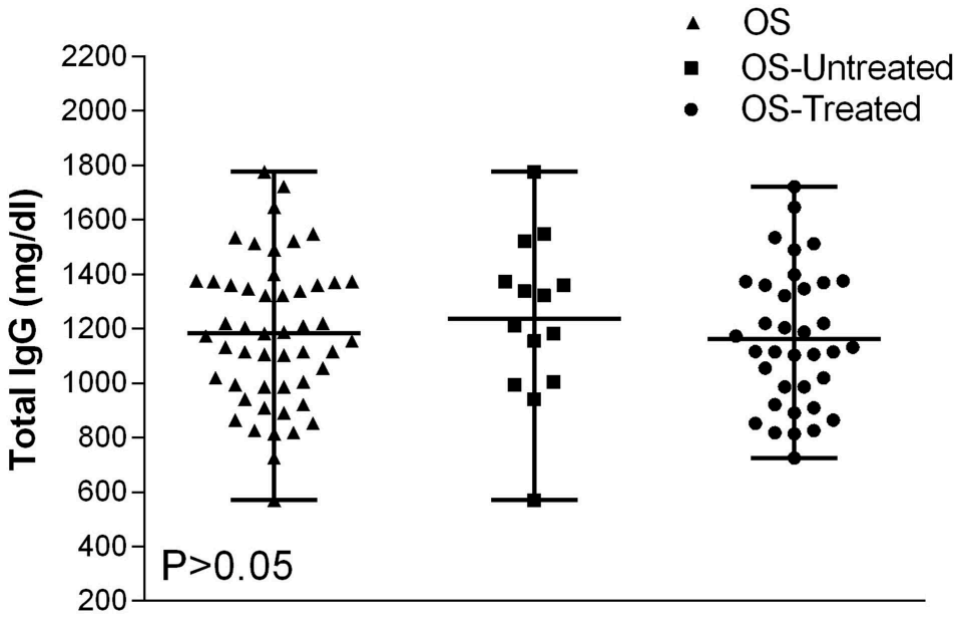
Total IgG variability values in serum samples from OS affected patients. Data are presented as IgG mean values and their ranges marked by short horizontal lines. The IgG levels present in serum samples of all OS patients are as follows: IgG = 1,182 mg/dL, mean value, which is in the range of 571–1,777 mg/dL; in untreated OS patients, IgG = 1,236 mg/dL mean value, range = 571–1,777 mg/dL; and in treated OS patients, IgG = 1,162 mg/dL, range = 726–1,722 mg/dL. All these IgG values are among normal concentrations, as reported for HS (Dati et al., 1996; Gonzalez-Quintela et al., 2008). Total IgG values detected OS patients were not statistically different from HS (*P* > 0.05), by Anova and Newman-Keuls multiple comparisons test.

The total immunoglobulin G (IgG) concentrations showed normal distribution in all osteosarcoma patients (Figure 4). IgG concentrations revealed in all OS patients (mean level 1,182 mg/dL, range 571–1,777 mg/dL) compared to IgG detected in OS untreated patients (mean level 1,236 mg/dL, range 571–1,777 mg/dL), and in OS treated patients (mean level 1,162 mg/dL range 726–1,722 mg/dL) were not statistically different (*P* > 0.05). Thus, the IgG concentration in sera from OS is similar to HS (Figure 4).

## Discussion

In this study, OS (*n* = 249) and HS sera (*n* = 247) with a similar median age were analyzed by indirect ELISAs for their reactivity to SV40 Tag mimotopes. The results of the investigation indicate that the overall prevalence of IgG antibodies against SV40 Tag in sera from OS patients and HS was 35 and 23%, respectively. These results suggest that in OS patients the prevalence of SV40 Tag serum IgG antibodies is significantly higher than in HS (^*^P < 0.05) (Barbanti-Brodano et al., 1998; Knowles et al., 2003; Viscidi et al., 2003). It has been reported that SV40 could be transmitted through contact in the home environment, as well as other communities (Tognon et al., 2016; Mazzoni et al., 2017a,c).

SV40 footprints were detected in different specimens of children and adults, such as blood, stool and urine, indicating that the SV40 spread may occur by distinct ways in the general population (Butel et al., 1999; Pancaldi et al., 2009; Vanchiere et al., 2009). Present data, on the prevalence of IgG antibodies reacting to SV40 T antigen, obtained herein with a large sample size, seem to confirm and extend the results reported in an earlier study on OS sera, which were found to be highly positive for IgG antibodies against SV40 structural protein antigens (VP 1-3 epitopes) (Mazzoni et al., 2015). Indeed the prevalence of serum IgG antibodies from OS patients reacting to SV40 T antigen (87/249 = 35%) revealed in this study does not differ compared to that of IgG antibodies reacting to SV40 VP epitopes (24/55 = 44%) (*P* > 0.05) detected in another cohort of OS patients, reported in a previous investigation (Mazzoni et al., 2015).

Altogether, these results indicate that OS patients might be more prone to be infected by SV40 than HS. In addition, indirect ELISAs were employed to assay IgG antibodies reacting to SV40 Tag from the cohort of BC affected patients, used as a control cancer, which is unrelated to SV40. It turned out that the prevalence of SV40 Tag antibodies detected in this cohort of BC affect patients did not differ significantly from HS. This result is important because it may indicate that malignancies in general do not influence the antibody response to SV40 Tag.

In our study, a large sample size of sera from patients affected by different types of OS were assayed by ELISA to verify SV40 Tag antibodies. Sera from conventional grade 4 OS, including osteoblastic (*n* = 193), chondroblastic (*n* = 20), and fibroblastic (*n* = 25) subtypes, as well as other OS (Table 1), did not show a significant different prevalence for SV40 Tag antibodies among them. Statistical analyses carried out in OS treated with chemotherapeutic drugs and OS untreated suggest that the SV40 Tag-prevalence of antibodies in their sera (34 vs. 37%) is not statistically different (*P* > 0.05), while it is statistically significant when both OS cohorts are compared to HS (^◦§^*P* < 0.05) (Table 2). Considering that sera from OS-treated and -untreated patients showed a similar prevalence of IgG antibodies against SV40 Tag, it is clear that drugs employed do not play a significant role in the immune system functions of OS patients, at least related to the production of specific IgG immunoglobulins against SV40 Tag antigen (Table 2, Figure 3).

Sera from OS, whether treated with chemotherapy or not, tested SV40 Tag-positive showing lower OD values than in HS. These results were obtained for the Tag A, Tag D, and both Tag A+D peptides (Figure 1). It is possible that the reduced ability of OS patients to respond to SV40 Tag antigens could depend on their oncologic status. It is known that the immune system of cancer patients is altered, at least in part. In our cohorts, the total immunoglobulin OD values determined in serum samples from OS and HS are similar. This result suggests that the higher prevalence of OD values for IgG against SV40 Tag analyzed in sera from OS vs. HS does not depend on a general impairment of OS immune system.

The development of OS, as in other malignancies, is due to many different gene alterations/mutations accumulating over time (Gianferante et al., 2017). At present, the specific causes/agents generating different DNA/chromosome aberrations are not completely known. In some studies, PCR amplicons belonging to SV40 were detected in human cancers, including OS, while other investigations did not reveal SV40 sequences in their tumor specimens. In addition, it has been published that some SV40-positive PCR data were obtained by contaminated reactions. To circumvent these conflicting PCR results, in our new studies the association between OS and SV40 was investigated by novel immunological approaches.

Our ELISA data with specific SV40 mimotopes, both from the VPs and Tag, indicate that this oncogenic polyomavirus might be responsible of the OS onset (Mazzoni et al., 2015). It is also possible that SV40 could simply act as a passenger virus replicating in tumor cells, while its multiplication has hampered in normal cells. Although our data indicate an association between OS and SV40, it is not yet known if this polyomavirus is involved in the human OS development. We can speculate that SV40 could act in children/young individuals when the function of their immune system is not fully mature due to age, or in the elderly when the immune system declines thus favoring, in the long run during the multistep process of tumorigenesis, the OS onset/progression.

## Ethics statement

The County Ethical Committee of Ferrara, Italy, approved the project, study number 151078.

## Author contributions

EM, MB, PP, FM, and MT designed the experiments. EM developed the methodology. EM, ET, MR, AS, and IB performed the experiments. EM, PR, MB, PP, MC, FM, and MT analyzed the data (e.g., statistical analysis). EM, FM, and MT writing and review of the manuscript. MT and FM supervision.

### Conflict of interest statement

The authors declare that the research was conducted in the absence of any commercial or financial relationships that could be construed as a potential conflict of interest.
